# Importance of Chemical Blanks and Chemical Yields in Accurate Trace Chemical Analysis

**DOI:** 10.6028/jres.093.025

**Published:** 1988-06-01

**Authors:** W. R. Kelly, S. A. Hotes

**Affiliations:** Inorganic Analytical Research Division, Center for Analytical Chemistry, National Bureau of Standards, Gaithersburg, MD 20899

## 1. Introduction

“The fundamental limitation to accuracy in chemical analysis is systematic error” [1]. Systematic error arises whenever the actual nature of the measurement process differs from that assumed. For a measurement to be both accurate and precise the measured value must be corrected for all sources of systematic error or bias and the true value must lie within the stated uncertainty with some stated level of confidence. Accurate chemical determinations require accurate knowledge of the chemical blanks and chemical yields at each stage of the separation process. A true blank correction can only be made if the exact functional form of the blank correction is known. If the blank correction is not exact, a bias may be introduced in the final value.

Murphy has noted that the analytical blank may be considered the “Achilles Heel” in chemical analysis [2]. Frequently, precise and otherwise accurate methods produce highly biased results from a lack of knowledge of, or improper consideration of, the chemical blank. For many types of measurements it is frequently necessary or highly desirable to isolate the element of interest in essentially pure form from the matrix in which it is found. This procedure typically involves decomposition of a sample with mineral acids and isolation of one or more elements by several solvent extraction or ion exchange steps. In each purification step, sample and blank atoms may be lost resulting in blank amplification in succeeding steps. The relationship between chemical yields and blank amplification is seldom discussed in the literature. Although blanks are frequently discussed in the chemical literature and guidelines for evaluating the blank correction have been published (see e.g., [3]), we are aware of only a few papers where the relationship between blanks and chemical yields is discussed [4–7].

In this note, we limit our focus to the effect of chemical blanks and chemical yields on the accuracy of chemical measurements. Their effect on both accuracy and precision will be discussed in more detail in a later paper.

## 2. Definition of Chemical Yield and Chemical Blank

For this discussion the term chemical yield is synonymous with the term recovery which refers to the fractional amount of a substance reclaimed from a purification process. We will take the following as an operational definition of the chemical blank: the chemical blank is the sum of all sources of the element or compound being determined that is not indigenous to the sample but is measured by the detector. Chemical species other than the analyte of interest that give the same response in the detector are not considered chemical blank.

## 3. Accuracy

An accurate measurement is one which obeys the following condition:

$$\left|M_{c}-T\right|=0$$ (1)

where *T* is the true value and *M_c_* is the corrected experimentally measured value given by one of the following two relations:

$$M_{c}=f\left(M,B_{i},Y_{j}\right)$$ (2a)

$$M_{c}=M-f\left(B_{i},Y_{j}\right)$$ (2b)

In actual practice we say that a measurement is accurate if it differs from the true value by less than some specified amount, say ϵ:

$$\left|M_{c}-T\right|<\epsilon$$ (3)

where *B_i_* and *Y_j_* are the chemical blanks and chemical yields which are subject to the boundary conditions:

$$b_{k}-<B_{i}<b_{l}$$ (4a)

$$y_{k}<Y_{j}<y_{l}$$ (4b)

where the absolute magnitude of the upper and lower bounds may or may not be equal. We are interested in examining the functional form [eqs (2a) and (2b)] that relates the measured value, the chemical blanks, and the chemical yields. In particular we wish to know under what conditions, if any, measurements can be made to obey eq (1).

## 4. Difference Between External and Internal Techniques

Chemical techniques can be grouped into two broad categories. For convenience, we will refer to techniques that have an internal standard as internal techniques whereas those that require external standards as external techniques. An example of an internal technique is isotope dilution in which an enriched or radioactive isotope of the element of interest is added to the sample. After dissolution of the sample and separation of the element of interest from the matrix the isotopic ratio of the mixture is then measured. Examples of the external techniques are the many spectroscopic techniques that compare the response of the sample to that of a standard. We wish to consider the functional form of several blank corrections and compare these to the exact blank corrections to ascertain the magnitude of the bias that is introduced. We will assume that measuring devices in both the internal and external cases are perfect and introduce no error. We will assume that both techniques must dissolve the sample and chemically separate the element of interest by an *n* -step separation process. During the dissolution step the sample picks up a reagent blank, *B*_R_, and at each successive separation step the sample is subject to a blank *B_i_*. After the chemical separation process the sample encounters an instrumental blank or loading blank inherent to the measurement system designated as *B*_L_. We will consider the case which is encountered in most instances in chemical analysis in which both sample and blank are lost together for a two-step (*n* =2) separation process.

### 4.1 External Case

For the external case the measured value is given in general by the following relation:

$$M=\left(T+B_{R}\right)\prod_{j=0}^{n}Y_{j}+\sum_{i=1}^{n}B_{i}\prod_{j=i}^{n}Y_{j}+B_{L}$$ (5)

which gives for *n* = 2 the following:

$$M=Y_{0}Y_{1}Y_{2}T+Y_{0}Y_{1}Y_{2}B_{R}+Y_{1}Y_{2}B_{1}+Y_{2}B_{2}+B_{L}$$ (6)

which on rearrangement gives for the true value the following:

$$T=\frac{M}{Y_{0}Y_{1}Y_{2}}-\left[B_{R}+\frac{B_{1}}{Y_{0}}+\frac{B_{2}}{Y_{0}Y_{1}}+\frac{B_{L}}{Y_{0}Y_{1}Y_{2}}\right]$$ (7)

Therefore, for the external technique eq (1) only holds true when the right hand side of eqs (2) and (7) are identical. It is evident from eq (7) that to measure the analyte accurately requires knowledge of eight unknowns. Note that the measured value must be divided by the total chemical yield before applying the blank corrections. Therefore, even if the chemical blanks are negligible the measured value will be less than the true value for yields less than unity.

### 4.2 Internal Case

For the internal case the measured value, *M*, is given in general by the following:

$$M=T+B_{R}+\sum_{i=1}^{n}B_{i}\prod_{j=0}^{i-1}Y_{j}^{-1}+B_{L}\prod_{j=0}^{n}Y_{j}^{-1}$$ (8)

which gives on rearrangement for *n*=2 the following for the true value, *T:*

$$T=M-\left[B_{R}+\frac{B_{1}}{Y_{0}}+\frac{B_{2}}{Y_{0}Y_{1}}+\frac{B_{L}}{Y_{0}Y_{1}Y_{2}}\right]$$ (9)

which is similar to eq (7) for the external case but with the very important difference that the measured value is not divided by the total chemical yield. Therefore, if the chemical blanks are negligible the measured value will be equal or very close to the true value. This is a unique advantage of isotope dilution compared to other techniques. It is commonly stated that the accuracy of isotope dilution is independent of chemical yields. However, it is important to emphasize that the chemical yield does in fact enter into the final value of an isotope dilution measurement in the blank correction terms as shown in eq (9). This fact is frequently over-looked or not considered when measurements are reported.

## 5. Comparison of External and Internal Methods

A simple way of illustrating the differences between the two methods is to consider the case which is often encountered in trace determinations where the blank approaches the size of the analyte of interest. For illustration let us assume that *T*= 10, *B*_R_=1, *B*_1_=2, *B*_2_=2, *B*_L_=0.1 and *Y*_0_=1.

### 5.1 Case A—No Blank or Yield Correction

**Figure 1:**
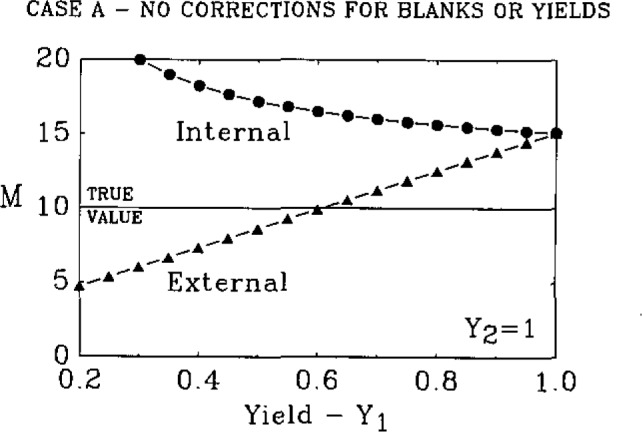
Plot of the measured values for the External [eq (6)] and the Internal [eq (8)] methods.

In figure 1, the measured values for the external [eq (6)] and internal [eq (8)] techniques are plotted assuming no corrections for chemical blanks or chemical yields. Note that the internal method is close to the true value but is biased in the positive direction for all values of *Y*_1_. Because *Y*_2_ only appears as a coefficient of *B*_L_, the measured value is not strongly influenced by the value of *Y*_2_ [see eq (8)]. The measured values from the external technique define a line of negative slope whose intercept is strongly influenced by the value of *Y*_2_. As *Y*_2_ becomes smaller the line moves to lower values.

### 5.2 Case B—Total Blank Correction, No Yield Correction

**Figure 2:**
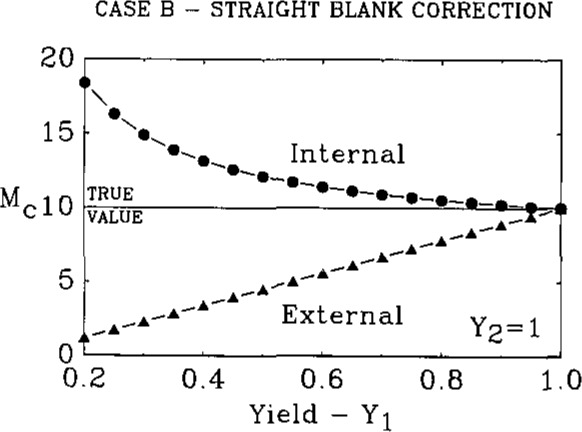
Plot of the corrected measured values [eqs (10) and (11)] using as the blank correction total subtraction of the chemical blank.

This is the type of correction that is frequently used by chemists and is referred to as a straight blank correction. In this case the total chemical blank, *B*_T_=*B*_R_+Σ*B*_i_+*B*_L_, is subtracted from the measured value, *M*. This gives for the external and internal techniques the following two equations which are plotted in figure 2 for *Y*_2_= 1:

External Technique
  $$M_{c}=T\left(Y_{0}Y_{1}Y_{2}\right)+B_{R}\left(Y_{0}Y_{1}Y_{2}-1\right)+B_{1}\left(Y_{1}Y_{2}-1\right)+B_{2}\left(Y_{2}-1\right)$$ (10)
Internal Technique
  $$M_{c}=T+B_{1}\left(\frac{1}{Y_{0}}-1\right)+B_{2}\left(\frac{1}{Y_{0}Y_{1}}-1\right)+B_{L}\left(\frac{1}{Y_{0}Y_{1}Y_{2}}-1\right)$$ (11)

From inspection of eqs (10) and (11) one can see that for the external case *M*_c_⩽*T* for all values of *B_i_*, whereas the converse (*M*_c_⩾*T*) is true for the internal case. As *Y*_2_ becomes smaller the external line drops to lower and lower values whereas the internal line is essentially unchanged due to the small dependence of eq (11) on *Y*_2_. It is clear from figure 2 that the internal method gives a more accurate result for all values of *B_i_* and *Y_j_*.

### 5.3 Exact Correction—Internal Case

It should be noted that with isotope dilution it is possible to measure the chemical yield at any point in the chemical separation for each sample by adding another isotope to the sample. For the case of *n* =2, if one knows the *B_i_*’s, and determines the chemical yield after step 1, this allows the following approximate blank correction to be used:

$$f\left(B_{i},Y_{j}\right)=B_{R}+B_{1}+\frac{B_{2}}{Y_{0}Y_{1}}+\frac{B_{L}}{Y_{0}Y_{1}}$$ (12)

Subtracting the r.h.s. of eq (12) from the r.h.s. of eq (8) gives the following for the corrected value:

$$M_{c}=T+B_{1}\left(\frac{1}{Y_{0}}-1\right)+B_{L}\left(\frac{1}{Y_{0}Y_{1}Y_{2}}-\frac{1}{Y_{0}Y_{1}}\right)$$ (13)

which for *Y*_0_=1 is a function of only *B*_L_ and the chemical yields. Since the coefficients of *B*_1_ and *B*_L_ are positive *M*_c_*⩾T*. Equation (13) yields values which differ from the true value by a very small amount. For example, for *Y*_1_=*Y_2_*=0.5 eq (13) equals *T* + 2*B*_L_.

## 6. Conclusions

The important point is that all approximations to the blank and yield correction introduce a systematic error into the final result whose magnitude will depend on the Sample/Blank ratio, the chemical yields, and blanks for individual steps in the separation process for *n* ⩾ 1. If chemical yields cannot be determined, they should be bounded. The number of separation steps should be kept as small as possible because the number of unknowns is equal to 2*n*+4. For *n*=2, a nearly exact solution can be used in isotope dilution determinations which is not true for the external method case because the yields must be determined in parallel experiments and cannot easily be determined on an individual sample.
